# XVII International AIDS Conference: From Evidence to Action - Basic science

**DOI:** 10.1186/1758-2652-12-S1-S3

**Published:** 2009-10-06

**Authors:** David Gilden, Rodney Kort

**Affiliations:** 1 David Gilden, New York, 10025, USA; 2 Kort Consulting, Toronto, M4Y 2T6, Canada

## Abstract

This article focuses on the sessions in which basic science research was presented at the XVII International AIDS Conference (AIDS 2008). It also provides an analysis of basic science issues which generated significant discussion and debate at the conference and are likely to have implications for future laboratory and clinical research. Data presented at AIDS 2008 confirmed the speed with which HIV establishes latent viral reservoirs following infection and the resulting challenges to viral eradication given how effectively HIV proviral RNA inserts itself into human DNA within these reservoirs. Studies also raised questions about the source of residual viremia and how these might be targeted by novel therapeutic approaches.

## Discussion

HIV has become a chronic, manageable disease for most patients in high-income countries, who can expect to live near-normal lifespans at current standards of care. Two major journal reports confirming this conclusion were released on the eve of the conference and brought a new focus to conference presentations on the basic biology behind the HIV-human host interaction [1,2].

### Residual viremia and the limits of HIV disease management

Several presentations at AIDS 2008 examined the limits of medical management of HIV. The virus can be reduced to levels undetectable by standard assays, yet a small residual amount of virus remains, on the order of 1 copy of HIV RNA per millilitre of plasma. The source of this residual viremia is still the subject of debate, but its consequence is certain. Patients who stop antiretroviral therapy (ART), even after many years, usually see their viral loads rebound to pre-treatment levels within a matter of weeks. Absent viral eradication, ART is for life. The world is therefore faced with the need to administer decades of antiretroviral agents to 30 or 40 million people, with all the expense and toxicity management issues that come with such a massive undertaking.

Robert Siliciano (Johns Hopkins University, USA) summarized a decade's worth of research in his presentation on the origin of residual viremia [3]. Siliciano's own observations have led him to infer that there is essentially no ongoing viral replication during successful ART. Instead, residual viremia comes from cells containing latent HIV DNA in their genomes. HIV becomes activated along with the cells' own genes during the immune response to disease.

There is no evolution in this latent population and thus no emergence of drug resistance. ART can control the residual infection indefinitely but it cannot eradicate it. More frustrating yet, the residual viremia in about half of all virologically suppressed patients seems cloned from a handful of isolates. Siliciano argues that on a few occasions, HIV becomes integrated into progenitor cells that faithfully copy the HIV genes as they divide and differentiate into mature immune cells such as monocytes and lymphocytes. Viral production begins only when these mature cells become activated.

In this context, the possibility of therapeutically eliminating all latent HIV or removing HIV that silently replicates along with the human genome in progenitor cell lines is remote. In a presentation, Anthony Fauci (National Institute of Allergy and Infectious Diseaes, USA) suggested a form of "immunotherapy" - in addition to early treatment and treatment intensification - as one strategy for gradually eliminating the latent reservoir [4]. While protected by the most potent ART, anti-HIV immune responses could be preserved and ultimately enhanced. Eventually, they might be strong enough, and residual HIV low enough, to allow for drug discontinuation without viral rebound.

The first step toward developing a therapeutic strategy for controlling latent HIV will be to understand more precisely how HIV proviral DNA integrates into human chromosomal DNA and the factors inducing latency. Kadreppa Sreenath (National Centre for Cell Science, India) presented data supporting suppression of transcription by SMAR1, a component of the cellular nuclear protein matrix; SMAR1 maintains the genes' physical structure as well as helping to regulate their activity [5]. SMAR1, induced in response to HIV infection, forms a repressor complex with two other proteins that bind to the long terminal repeat region at the terminus of the HIV genome. HIV tat, together with the cells' own activation factor NF-κB, displaces this complex and triggers viral replication. This model yields hints of a therapeutic strategy, involving either SMAR1 promotion or tat inhibition, but this concept is still far from a concrete therapeutic application.

### The challenging speed of acute infection

The conference yielded multiple presentations of how rapidly HIV takes over during acute infection. Eric Hunter and Debrah Boeras (Emory University, USA) showed that new sexually acquired HIV is usually very genetically homogenous [6,7]. It arises from a minor variant present in the donor's body. Presumably such HIV has special, still undefined characteristics that make it more fit for transmission.

Once in contact with a new host, HIV moves very quickly. Yonatan Ganor (Institut Cochin, France) described the results from his group's explant model of human foreskin. [8] Cell-associated HIV was efficiently transmitted in this model. The Langerhans cells on the inner foreskin became infected with HIV and transferred the virus to the CD4+ T-cells in the dermis within one hour of initial contact. In contrast, transport from the outer foreskin was 10 times less efficient. Cells in the outer foreskin's keratinized layer could become infected but that layer kept the infection from spreading inward. Also, the Langerhans cells in this layer degraded HIV when they captured it rather than transporting it live to the virus's primary target.

**Figure 1:**
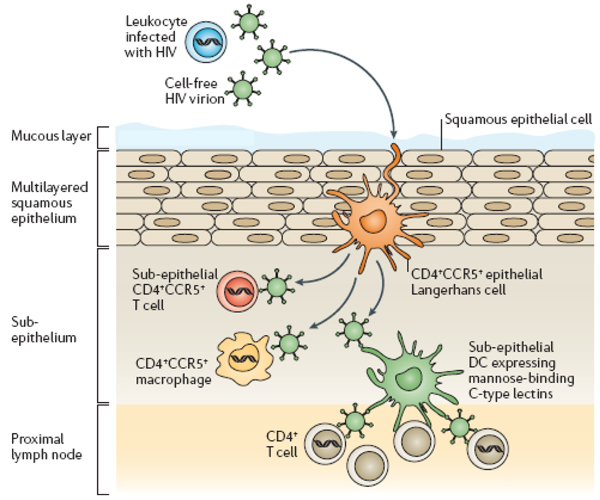
**Cells Playing a Role in Genital HIV Transmission**. Source: Vanham, G. Future promising microbicidal products: What to learn from the in vitro work (THSY0601), from Lederman, M Offord, R and Hartly O. Microbicides and other topical strategies to prevent vaginal transmission of HIV. Nature Reviews Immunology 6, 371-382 (1 May 2006)

The role of mucosal dendritic cells, of which Langerhans cells form a subset, in facilitating HIV infection has been known for some time (Figure 1). Dendritic cells contain surface receptors containing C-lectin, to which HIV physically adheres [9]. DC-SIGN is the most widely recognized of these receptors, but there are a number of others.

Dendritic cells' normal function is to present foreign antigen to the CD4+ T-cells, which then stimulate an immune response. The dendritic cell first internalizes the C-lectin receptor-HIV complex in an endosome. Endosomes normally break up foreign bodies for antigen presentation. Whether particular dendritic cell subsets actually degrade or protect HIV depends on the structure of their C-lectin receptors. This observation may help explain HIV's different outcome on the inner and outer foreskin - and hence the protective effect of circumcision [10].

Tove Kaldensjö (Karolinska Institute, Sweden) reported on four different dendritic cell subsets present on the female ectocervix [11]. Comparing HIV-negative women with high and low risk for HIV exposure, Kaldensjö's team found that the women with higher risk sexual behaviour had more ectocervical dendritic cells with C-lectin receptors capable of transporting HIV to lymphoid tissue.

Damage occurs very quickly once HIV starts infecting CD4+ T-cells. Fauci noted that HIV establishes a latent HIV reservoir in the first week after transmission. In addition, gastrointestinal effector CD4+ T-cells are virtually eliminated during the first few weeks [12,13]. According to a recent report by Fauci's group, CD4+ cell counts in gut lymphoid tissue remain depressed even after 10 years of suppressive ART, and this tissue represents the location of most of the latent viral reservoir [14].

### Understanding innate immunity and the role of toll-like receptors

Halting HIV's rapid progress during primary infection is of prime importance. AIDS 2008 marked a new interest in innate immunity, which is the first line of defence against HIV. The initial inflammatory response arises from a variety of non-specific, T-cell independent immune responses that recognize invasion by some sort of foreign material. HIV's encounter with innate immunity starts with the C-lectin receptors on mucosal dendritic cells. Though mostly ignored until recently, innate immunity remains a major factor throughout the HIV lifecycle.

Much of the new focus on innate immunity concerns toll-like receptors (TLRs). TLRs are an ancient family of cell-surface and internal receptors. They recognize molecules that are common to many pathogens but foreign to host cells. Present on human immune cells, including macrophages and lymphocytes, each receptor in the family specializes in recognizing a certain type of molecule - for example, bacterial glycolipids or lipoproteins and viral DNA or RNA. Once excited, TLRs start a signal cascade that results in cell activation and the release of inflammatory cytokines.

Research into the relationship between TLRs and HIV has accelerated dramatically over the past five years. The AIDS 2008 Scientific Programme included several inconsistent, and even conflicting, reports on strategies to employ TLRs therapeutically.

**Figure 2:**
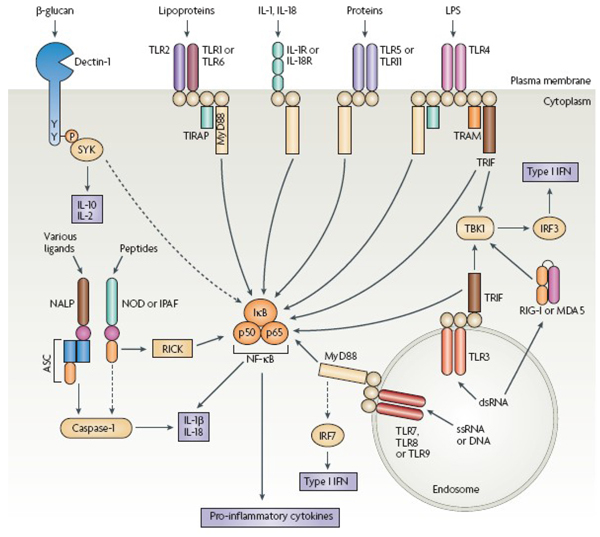
**The Toll-like Receptors in and on Cells Recognize Microbial Patterns and Trigger Immune Responses**. Source: Pimenta-Inada, H *et al.* The Toll-like receptor 2 ligand Zymosan inhibits HIV-1 replication in human primary cells (MOAA0105), from Trinchieri G., Sher A., Nature Reviews Immunology March 2007, 7(3): 179-90

Dumith Bou-Habib (Oswaldo Cruz Institute, Brazil) reported on zymosan, a common fungal polysaccharide that binds to TLR2. Zymosan inhibits HIV infection in macrophages, apparently at the cell entry stage (Figure 2) [15]. A synthetic compound, Pam3Cys has similar properties. However, Sandra Thibault (Research Centre in Infectious Diseases, Canada) reported that Pam3Cys and other compounds binding TLR2 and TLR5 on CD4+ T-cells actually increase cellular HIV integration and production, a result that conflicts with Bou-Habib's findings [16]. Terrance Brann (SAIC-Frederick, Inc., USA) also presented findings suggesting the HIV suppressive effects of TLRs. His experiments were with a pair of TLR4 ligands produced by human neutrophils [17]. These molecules reduced R5-tropic HIV replication in macrophage cultures but did not affect X4-tropic HIV in CD4+ T-cells. In other research, Leonid Margolis (National Institute of Child Health, USA) discussed ways in which viral co-infections such as HCV can up- and down-modulate HIV, probably by interacting with various TLRs [18].

The inconsistencies in the studies presented require further investigation. One explanation may be subtle ways in which the TLR-signalled response directs chemokine and cytokine release. The zymosan report, for example found that macrophages respond to TLR2 stimulation by releasing beta-chemokines, the chemotactic signalling molecules that fit into and block the CCR5 receptor utilized by R5-tropic HIV when entering new cells. T-cells respond to TLR2 by producing NF-kB, as do macrophages, but without the beta-chemokines.

The importance of the overall cell-signalling milieu was further stressed in a report published just after the conference. It described the HIV suppressive effect of a mutation in TLR8 [19]. The study, which included 782 HIV+ patients, observed that those with the mutant (A1G) gene in TLR8 exhibited a mean CD4 decline that was 3.5-fold slower than HIV+ patients with normal TLR8 (75% of the total study population).

When stimulated, the mutant TLR8 triggers relatively lower levels of NF-κB and IL-10 and relatively higher levels of tumour necrosis factor alpha (TNF-α) as compared with normal TLR8. All three are known to promote HIV replication, but TLR8 stimulation and TNF-α also activate protective CD8+ cytotoxic lymphocytes and natural killer (NK) cells [20].

NK cells are a type of white blood cell that non-specifically kills virus-infected cells. At AIDS 2008, Samuel Nuvor (University of Nairobi, Kenya) and colleagues argued that down-regulation of NK cells is a critical difference between HIV-1 infection and HIV-2, which progresses more slowly [21]. Restoring NK cell activity via TLR8 or otherwise might be an important component in therapeutically or prophylactically creating an effective immune response against HIV.

### Harnessing the immune response

Stimulating the immune system to better fight HIV is a complicated issue. Chronic immune system activation without achieving effective HIV control may be a major contributor to HIV-associated T-cell loss [22]. Immune control and regeneration processes become exhausted or dysfunctional. They fail to replace cells lost to HIV or even cause more cells to die via a form of cell suicide known as apoptosis. A number of the factors contributing to immune decline were described at the conference. These include loss of proliferative capacity and response to antigen presentation in HIV-specific CD8+ cytotoxic lymphocytes [23,24].

Notably, a post-AIDS 2008 report described increased cellular TLR levels and heightened responsiveness to TLR signaling in persons with HIV [25]. This report implicates TLRs in immune dysfunction during untreated HIV infection rather than as a source of protection.

Many of the conference presenters nonetheless suggested that their research findings will eventually help to identify ways to restore the immune response and devise new means for controlling HIV. The relevance of these results is not yet clear given the high degree of HIV control achieved by direct antiretroviral therapy. Suppressing HIV will by itself eliminate much of the chronic inflammation and allow immune recovery. The total CD4+ T-cell count does not seem to ever return to pre-HIV levels, however, and subtle defects in immune subpopulations remain [26].

An obvious next step will be to better delineate what constitutes effective anti-HIV immunity. Answering that question is complicated by the fact that the behaviour of individual immune components can have both positive and negative effects. As the inconsistent TLR findings discussed previously suggest, researchers need to consider how each component interacts with other aspects of the immune system. Considering that HIV disease feeds on immune activation, the ultimate goal is a plan for deploying the various immune defences to provide maximum effectiveness with the least extraneous activity.

Manipulation of the immune system may eventually prove useful in further restoring the immune system after the antiretroviral agents have reduced HIV to undetectable levels. In particular, enhancing the anti-HIV immune response promises to help block residual HIV, perhaps allowing for simplification or elimination of drug therapy. In addition, immune therapy may prove more effective before the body ever comes in contact with HIV. Vaccines and other preventive technologies have so far been unable to block HIV transmission. Selectively stimulating appropriate immune responses could prove vital to advancing such prevention efforts.

## Conclusion

The complexity and dynamism of HIV pathogenesis and host/virus dynamics continue to present both new challenges and new opportunities for therapeutic interventions. Studies at AIDS 2008 advanced our understanding of the role of TLRs and other cellular mechanisms in the inflammatory response to HIV, possible avenues for manipulating the immune response to better control HIV pathogenesis, and how the speed of acute infection and ongoing residual viremia continue to present barriers to the viral eradication. Conflicting data on the role of TLRs in up or down-regulating HIV expression, and debate regarding the source of residual viremia and potential strategies on how to deal more effectively with viral reservoirs are important issues for the field that will need to be addressed in future research.

## Competing interests

David Gilden and Rodney Kort are independent consultants contracted by the International AIDS Society for the purpose of preparing the AIDS 2008 Impact Report for publication.

## Authors' contributions

David Gilden drafted the initial text and Rodney Kort provided editorial input and advice. Both authors have approved the manuscript for publication.
